# *Ex vivo*, *in situ* perfusion protocol for human brain fixation compatible with microscopy, MRI techniques, and anatomical studies

**DOI:** 10.3389/fnana.2023.1149674

**Published:** 2023-03-23

**Authors:** Ricardo Insausti, Ana María Insausti, Mónica Muñoz López, Isidro Medina Lorenzo, Maria del Mar Arroyo-Jiménez, María Pilar Marcos Rabal, Carlos de la Rosa-Prieto, José Carlos Delgado-González, Javier Montón Etxeberria, Sandra Cebada-Sánchez, Juan Francisco Raspeño-García, María Mercedes Iñiguez de Onzoño, Francisco Javier Molina Romero, Ruth Benavides-Piccione, Silvia Tapia-González, Laura E. M. Wisse, Sadhana Ravikumar, David A. Wolk, Javier DeFelipe, Paul Yushkevich, Emilio Artacho-Pérula

**Affiliations:** ^1^Human Neuroanatomy Laboratory, Neuromax CSIC Associated Unit, Medical Sciences Department, School of Medicine and CRIB, University of Castilla La Mancha, Albacete, Spain; ^2^Department of Health, School of Medicine, Public University of Navarra, Pamplona, Spain; ^3^Laboratorio Cajal de Circuitos Corticales, Centro de Tecnología Biomédica, Universidad Politécnica de Madrid, and Instituto Cajal, CSIC, Madrid, Spain; ^4^Department of Diagnostic Radiology, Lund University, Lund, Sweden; ^5^Department of Radiology, University of Pennsylvania, Philadelphia, PA, United States; ^6^Department of Neurology, University of Pennsylvania, Philadelphia, PA, United States

**Keywords:** human brain, fixation, carotid perfusion, histology, electron microscopy, intracellular injection, MRI

## Abstract

We present a method for human brain fixation based on simultaneous perfusion of 4% paraformaldehyde through carotids after a flush with saline. The left carotid cannula is used to perfuse the body with 10% formalin, to allow further use of the body for anatomical research or teaching. The aim of our method is to develop a vascular fixation protocol for the human brain, by adapting protocols that are commonly used in experimental animal studies. We show that a variety of histological procedures can be carried out (cyto- and myeloarchitectonics, histochemistry, immunohistochemistry, intracellular cell injection, and electron microscopy). In addition, *ex vivo*, *ex situ* high-resolution MRI (9.4T) can be obtained in the same specimens. This procedure resulted in similar morphological features to those obtained by intravascular perfusion in experimental animals, provided that the postmortem interval was under 10 h for several of the techniques used and under 4 h in the case of intracellular injections and electron microscopy. The use of intravascular fixation of the brain inside the skull provides a fixed whole human brain, perfectly fitted to the skull, with negligible deformation compared to conventional techniques. Given this characteristic of *ex vivo*, *in situ* fixation, this procedure can probably be considered the most suitable one available for *ex vivo* MRI scans of the brain. We describe the compatibility of the method proposed for intravascular fixation of the human brain and fixation of the donor’s body for anatomical purposes. Thus, body donor programs can provide human brain tissue, while the remainder of the body can also be fixed for anatomical studies. Therefore, this method of human brain fixation through the carotid system optimizes the procurement of human brain tissue, allowing a greater understanding of human neurological diseases, while benefiting anatomy departments by making the remainder of the body available for teaching purposes.

## Introduction

The human brain has many unique features that are quite different from those of other primates, and even more different if we consider rodents. Investigating the functional significance of brain regions and networks often involves direct examination of human brain tissue.

For these reasons, procurement of good quality brain tissue obtained at autopsy is an important and necessary step in basic and clinical neuroscience, yet its availability is rather limited. Human brain tissue is a precious resource that should be used carefully to sustain the progressive understanding of human brain structure in normal and pathological circumstances.

Different studies of human brain morphology—from gross anatomy and fiber bundle dissection (Klingler and Gloor, 1960) to electron microscopy—involve different methodological problems, which makes it difficult to apply a variety of anatomical methods in the same brain tissue (for review, see Grinberg et al., 2007; McFadden et al., 2019). The best preservation of human brain structures (as in other species) is determined by the optimization of processes to slow the breakdown of cellular integrity. To a large extent, slowing or stopping the deterioration of brain tissue after death involves different factors. The first and most critical is the postmortem interval (PMI), which is the time elapsed between death and the start of brain fixation. Ideally, the PMI should be as short as possible to try to obtain brain tissue with a quality like that obtained in experimental animals, in particular nonhuman primates (Lavenex et al., 2009; Blair et al., 2016).

The second fundamental factor is the actual contact of brain tissue with the fixative. Many different brain fixation methods have been tried over the years for fixation of human brain tissue (Beach et al., 1987; Waldvogel et al., 2006; Grinberg et al., 2008; Alkemade et al., 2018, 2020; Maranzano et al., 2020). However, it should be noted that very few studies deal with the possibility that human brains are coming from human body donor programs (McHanwell et al., 2008), a significative source of whole human brains, as opposed to clinical or forensic autopsies, which are the major providers of human brain tissue.

The procurement of human brain tissue from body donor programs poses several additional difficulties. First, the use of body donations should abide by ethical rules for the use of bodies, or body parts, destined for teaching and scientific research, after appropriate informed consent is signed by the donor or next of kin. Second, specific national and regional regulations add further variability to the process of procuring the body, some of which are of primal importance for neuropathological and neuroscientific research (McHanwell et al., 2008).

Body donations and autopsies provide the means to establish brain banks which are a primary source of brain tissue for many studies. However, the usual procedure of using one hemisphere for fixation by immersion in formalin, and the other hemisphere for freezing and storing for biochemical, molecular biological, or genetic studies, makes it difficult to obtain whole brains for comparisons between hemispheres. The necessary removal of multiple samples for neuropathological diagnosis often limits the amount of tissue left for researchers. As a result, there is a clear need to obtain the maximal benefit from any given human brain donation. A new approach has been recently reported by McKenzie et al. (2022) for *ex situ* vascular fixation of the brain which maintains histological quality and *in situ* hybridization. Moreover, *ex vivo* MRI scans can be obtained.

Anatomy departments are well suited as a source of human brain tissue because they receive body (and brain) donations through willed body donor programs. The use of human bodies is a fundamental component of teaching gross anatomy to medical students and related health sciences. These programs have existed in the medical academic setting for almost two centuries in Schools of Medicine. The human brain and body donation programs are of common use in all Schools of Medicine in Spain. Their ethical approval derives from the signed, informed consent required for educational and research purposes. In this work, ethical approval was given from the University of Navarra (Spain, 1985–1998), and the University of Castilla-La Mancha (Spain) from 1998 to the present. Further information is available at the webpage: https://www.uclm.es/Albacete/Medicina/Facultad/programadonacioncuerpos).

Those programs also allow access to human brains for research purposes in addition to education. Brains from this source can be extracted swiftly and placed in fixative within a short PMI. Thus, anatomy departments are in a privileged position to overcome limitations associated with the restricted availability of brain tissue of brain banks, since is possible to fix brains via the same process used to embalm human bodies. Moreover, a recent report by Frigon et al. (2022) shows that mice perfused with several different fixatives used in embalming bodies retained antigenicity, allowing immunoreactivity to several antibodies to be demonstrated.

Another aspect that has arisen recently is the increasing number of neuroimaging studies that focus on normal or pathological structures in fixed brains. Neuroimaging studies have become more numerous over the last decade, attempting to determine the best correlation between pathology and MRI, both *in vivo* (Wisse et al., 2014, 2016; de Flores et al., 2020) and *ex vivo* (Augustinack and van der Kouwe, 2016; Adler et al., 2018; Beaujoin et al., 2018; Frigerio et al., 2021; Yushkevich et al., 2021). High-resolution neuroimaging exploration of the brain *ex vivo* requires fixation of the human brain compatible with radiological imaging procedures. The validity of identification and segmentation of brain structures in *ex vivo* and *in vivo* requires the best possible alignment between MRI and histology, in which anatomical boundaries can be inferred based on cytoarchitecture, as seen in *ex vivo* studies (Wisse et al., 2017; Adler et al., 2018; Iglesias et al., 2018; Yushkevich et al., 2021). Some groups have addressed the need for MRI-histology correlation by obtaining MRIs of patients or donors after death, without manipulation of the brain at all (*ex vivo, in situ* MRI studies (Frigerio et al., 2021). However, such imaging requires suitable facilities, in many cases restricted to hospitals, and rapid handling of the body, two reasons for this only being possible in a few centers (Frigerio et al., 2021). Furthermore, the time available to scan the brain before removal and fixation is limited, which in turn limits the resolution of *ex vivo, in situ* MRI studies.

*Ex vivo* MRI examination of fixed human brains is usually based on specimen immersion-fixed in 10% formalin, as has been used by us and others (Insausti et al., 1998; Franko et al., 2014). However, one of the main drawbacks of this procedure is the change in brain shape due to gravity when the brain is placed in a container with the fixative, which produces well-known ill effects on cortical and subcortical structural deformation (Maranzano et al., 2020).

Deformation may negatively affect the data obtained in different studies, such as brain atlases derived from *ex vivo* brain MRI (Maranzano et al., 2020). These authors report up to five different drawbacks for the neuroimaging of brains fixed *ex situ* (outside the skull) ranging from deformation of the brain to fixation gradient. However, although Maranzano’s recommendations are useful for MRI studies (Maranzano et al., 2020), further morphological investigation—through, e.g., immunohistochemical studies—may be virtually impossible, in part due to the overheating of brain tissue when detailed MRI anatomy requires a long time of exploration.

Morphological studies of the human brain need to overcome the caveat of an excessive PMI, which is very difficult for the researcher to control, mainly due to two variables: the swiftness with which funerary directors move the donor body to the anatomical or medical facilities, and the time required to prepare the body to remove the brain and place it in fixative. Coordination with the funeral directors regarding logistics is critical when the distance from the site of death may increase the PMI. The sum of these two variables often results in the PMI being close to 24 h.

While a good PMI is considered to be below 24 h (Maranzano et al., 2020), many studies surpass this PMI (Waldvogel et al., 2006; Blair et al., 2016). Some reports explore the rate at which RNA in nervous tissue deteriorates as a measure of brain degradation (Sherwood et al., 2011; Gonzalez-Riano et al., 2017). An additional factor to be considered is the delay until brain fixation is carried out.

Basic morphological studies (e.g., Nissl stain) can support a PMI longer than 24 h. However, Mouritzen Dam (1979) notes pyknosis in nuclei and the presence of “dark neurons” after 24 h of PMI. Lavenex et al. (2009), in a study conducted in nonhuman primates under controlled conditions, report changes in morphological features such as axonal coiling and decreased immunoreactivity for several different substances, concluding that beyond 24 h, significant morphological changes can be perceived in brain tissue. Nevertheless, Blair et al. (2016) report that, even with a PMI of up to 50 h, tissue maintains successful immunoreactivity for several proteins. There is no doubt that the use of immunohistochemical methods to characterize the chemical phenotypes of neurons and glia (Cuello, 1993) has provided major advances in the characterization of various substances in neurons and glia of the human brain. However, metabolomic and anatomical techniques have addressed possible PMI-induced changes and have shown significant metabolomic changes at 2 h PMI, whereas the integrity of neurons and glia, at the anatomical/neurochemical level, was not significantly altered during the first 5 h PT for most histological markers (Gonzalez-Riano et al., 2017).

It is beyond the scope of this report to deal with the numerous examples of immunohistochemistry performed in the human brain. Most of these studies used immersion fixation of the brain, with only a few using intravascular fixation (Beach et al., 1987; Insausti et al., 1995; Waldvogel et al., 2006; Alkemade et al., 2020). While immunohistochemical processing of human brain fixed by immersion provides good quality staining (which depends largely on the PMI, quality of the fixation and the type of substance to be visualized), we aimed at showing that the protocol we propose (under the name “**Neuromax**”) has the capacity to provide histological quality of human nervous tissue after different processing methods, as well as postmortem MRI, as detailed as the best immersion-fixed material—by allowing the brain to be in more direct contact with fixatives and an improved uniformity across cases. In short, the aim of our method is the adaptation of fixation protocols in animal studies to the human brain.

A further step in the study of human cell types is the intracellular filling of neurons and glia, a refinement related to the classical Golgi silver impregnation method. Intracellular filling results in the best possible material to perform morphometric analyses of different neuronal aspects such as the quantification of dendritic spines in normal and pathological conditions (Belichenko et al., 1994; Benavides-Piccione et al., 2020). Likewise, a comparative study of homologous regions of the brain in different species can be accomplished (Benavides-Piccione et al., 2020). In such cases, a short PMI is critical for the preservation of the most peripheral axonal or dendritic branches, facilitated by rapid contact between brain tissue and fixative. To date, the best method other than the collection of the peripheral tissue surrounding a lesion obtained immediately after neurosurgical resection (Belichenko et al., 1994) is intravascular perfusion, although direct immersion in the fixative is also valid, at least for superficial structures (e.g., cortical areas).

Electron microscopy techniques are particularly demanding with regard to the quality of fixation of the tissue (Hayat, 1981; Zemmoura et al., 2016; Lewis et al., 2019; Sele et al., 2019; Cano-Astorga et al., 2021), and the PMI is the most critical factor in ultrastructural studies (Sele et al., 2019). Brain banks propose a maximum of 16 h for the postmortem delay (Sele et al., 2019). However, at the higher end of the range reported, the PMI can be up to several days [100 h (Kay et al., 2013); 89 h (Henstridge et al., 2015); 50 h (Blair et al., 2016); 50 h (Krause et al., 2016)].

Modern neuroimaging studies bring together anatomy and neuroimaging, so different brain structures can be detected by MRI after proper identification of anatomical boundaries in histology, e.g., using cytoarchitectural features in Nissl sections. *In vivo* MRI research is jeopardized by the use of manual or automated segmentation protocols that overly rely on geometrical rules, and which may vary from laboratory to laboratory, and/or do not capture the full extent of anatomical variability (Yushkevich et al., 2015; Wisse et al., 2016, 2021a). By matching histology (particularly serially acquired histology) to *ex vivo* or antemortem *in vivo* MRI scans, it is possible to generate examples of MRI segmentations that are validated by cytoarchitectural information (Adler et al., 2018; Iglesias et al., 2018; Ravikumar et al., 2021; Casamitjana et al., 2022). This approach can be used to devise better manual MRI segmentation protocols or to train automated MRI segmentation tools. Such studies benefit from brain tissue with optimal fixation and minimal deformation between antemortem *in vivo* MRI, *ex vivo* MRI, and histology series.

Ensuring appropriate fixation is an important first step when preparing tissue for any histological analysis. Fixation of the donated body through the vascular system, either by the carotid or the femoral arteries, is the standard procedure in most anatomy departments. However, the solutions used for body fixation often preclude histological processing of the brain (see Frigon et al., 2022). Despite anatomy departments receiving numerous body donations, most of these bodies are not fully exploited when it comes to human brain studies. This situation prompted us to look for a suitable procedure allowing serial histology, immunohistochemistry, intracellular filling, and electron microscopy, as well as *ex vivo* MRI, in one single method of brain fixation by intravascular perfusion, that at the same time preserves the body for educational purposes such as anatomical teaching or the practice of surgical skills.

## Materials and methods

Human brains are obtained through our “Body and Brain Donation Program” at the University of Castilla-La Mancha as stated before. Informed and signed consent was obtained in all cases. Furthermore, the Ethical Committee of the University of Castilla-La Mancha has approved different research projects involving Human Neuroanatomy Laboratory (HNL) cases perfused through the carotids.

The intravascular-intracranial fixation of the human brain has been routinely carried out in the HNL since 2012. Over this period, we have collected 58 cases (age range: 37–97 years, mean: 69.5 years, 25 Males/21 Females). The PMI ranged between 1 h and 24 h, with a mean of 7.3 h. Three additional cases had the brain removed and fixed by immersion for comparative purposes. In addition, we had three more cases processed in the HNL at the Department of Anatomy, University of Navarra (Pamplona, Spain) by the authors AMI and RI, in which the brain was perfused after extraction from the skull *(ex vivo, ex situ)*. The method of *ex situ* vascular fixation, described in Insausti (Insausti, 1992) and recently employed by McKenzie et al. (2022)^1^.

### *Ex vivo*, *in situ* perfusion protocol

The protocol for human brain fixation in the HNL was first implemented in 1985 and has undergone gradual modifications while maintaining the basic purpose of fixing the human brains as closely as possible to the procedures used in the fixation of experimental animals. Experiments with nonhuman primates throughout this time provided the basis of the methodology applied to human brain fixation (Insausti et al., 1987; Blaizot et al., 2010). The basic fixative agent has always been 4% paraformaldehyde in 0.1 M phosphate buffer.

### Preparation of the dissection field

The body arrives directly from the death location at the anatomy facilities of the Albacete School of Medicine (University of Castilla-La Mancha, Spain), it is placed on an autopsy table in a prosection room, upon which the technical procedures are carried out. Perimetral aspiration of the autopsy table, as well as room aspiration of fixative fumes, is available to overcome excessive concentrations of formaldehyde in the environment. The ventilation system has probes that measure the concentration of formalin fumes and automatically sets aspiration when the concentration of formalin exceeds 0.003 ppm (parts per million), thus creating a safe atmosphere for breathing while performing the perfusion protocol.

**Figure 1:**
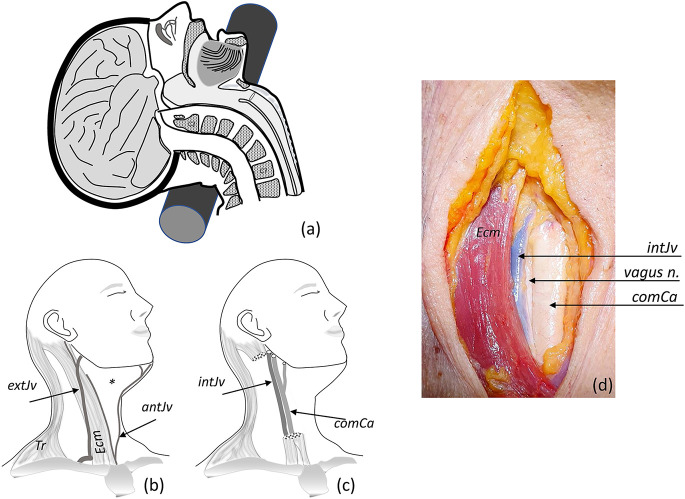
Fixation procedure. **(A)** Hyperextension of the neck using a cylindrical piece placed at the nuchal level (also occurs when the head hangs down from the edge of the table). **(B)** A slight turn of the head to the side, exploring neck superficial structures (*Tr*, trapezius muscle; *Ecm*, sternocleidomastoid muscle; external *extJv* and anterior *antJv* jugular veins and their connections); the asterisk marks the anterior triangle of the neck. **(C)** *Ecm* and superficial veins were removed for identification of the carotid sheath, identifying internal jugular vein *intJv*, and the common *comCa*, internal and external carotid arteries (the vagus nerve—not showing—lies behind the vascular structures). **(D)** Anterior view of the neck showing open carotid sheath (*comCa*, *intJv* retracted, and the vagus nerve).

Once the body has been placed in a supine position and the neck hyperextended, the head hangs from the table simply by letting it drop, thereby ensuring that the neck is in the best position to expose both carotid arteries (Figure 1A). If the neck were flexed, access to the carotids would be difficult. Furthermore, obese bodies with a fatty neck are difficult to dissect, an issue which can be largely overcome in this body position. Moreover, as the head hangs over a sink, blood and perfusion liquids can be collected in containers and disposed of appropriately.

Afterwards, the face is covered with a drape attached to the forehead that leaves the dissection field free. The surface anatomy of the neck is palpated to identify the anterior border of the sternocleidomastoid muscle (the big stretch of muscular flesh between the mastoid process, behind the ear, and the medial part of the clavicle). Thus, we identify the anterior triangle of the neck, which is limited by the neck’s midline, the inferior border of the jaw, and the anterior border of the sternocleidomastoid muscle. The midline of the neck is marked in some bodies (Adam’s apple), while it is not so prominent in females and needs to be located through palpation of the larynx (Figure 1B).

### Dissection of the neck

The skin is opened with a scalpel (preferably a number 4 handle with a 24-type surgical blade), and an incision is made between the lower border of the jaw and the clavicle’s midline. The borders of the initial opening are separated with the hand and an incision is made on the superficial layer of the investing cervical fascia (a whitish layer of connective tissue, sometimes traversed by cutaneous nerve branches of the supraclavicular nerves, which can be severed). The fascial sheath is opened with a longitudinal incision, medial to the anterior border of the sternocleidomastoid muscle (a thin layer of muscle, the platysma muscle, may lie superficial to the superficial cervical fascia). The pretracheal lamina, which fills the space under the sternocleidomastoid muscle with connective tissue, is opened by blunt dissection, while the pretracheal musculature (which extends longitudinally in the midline and is made up of sternothyroid and sternohyoid muscles) remains preserved 2–3 cm from the midline. The omohyoid muscle can be seen laterally to the pretracheal musculature, covering the access to the carotid sheath; this muscle can be cut and retracted.

In the approach to the carotid sheath, some bleeding may occur if superficial small veins in the lateral surface of the neck are cut. In most cases, this bleeding is limited and can be controlled by suction or gauze pressure. However, in some subjects, it stains the tissue and may obscure the exposure field. Nevertheless, the deep location of the carotid and jugular vessels does not prevent these vascular structures from being reached.

### Dissecting approach to the common carotid (before the division into internal and external carotids)

Next, the carotid sheath (connective tissue holding together the carotid artery, the jugular vein, and the vagus nerve) is identified and opened by blunt dissection of its contents (Figures 1C,D). In this step, it is important to carefully separate the carotid artery from the jugular vein. Special care must be taken to avoid puncturing the jugular vein (heavy bleeding results if the fine jugular wall is punctured). This would make the cannulation of the carotid artery difficult.

**Figure 2:**
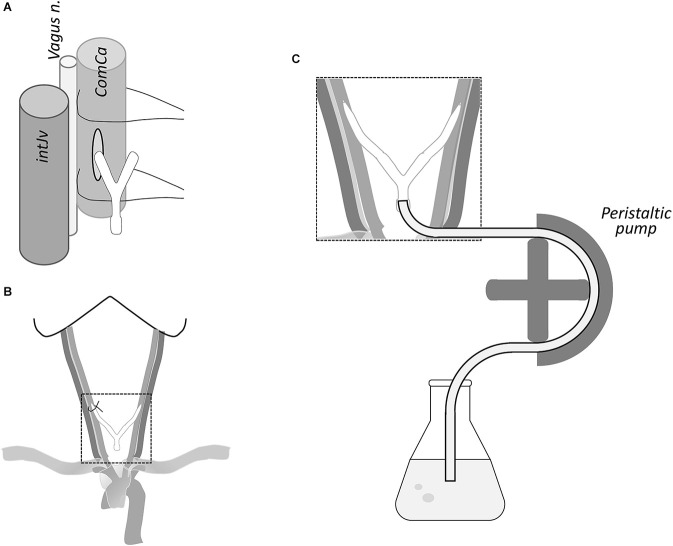
**(A)** Neurovascular component of the carotid sheath; a small incision of the carotid artery was made for insertion of the cannula. **(B)** Both neck cannulae linked by a Y tube are shown inserted in the carotid arteries with a surgical thread to fix the cannulae. **(C)** Peristaltic pump is used through the “Y” connector to introduce perfusion solutions (see text).

The morphological features of the carotid artery (thick, rounded wall, white or yellowish color, sometimes with arteriosclerotic plaques) facilitate separation from the surrounding tissue. Once selected, a double thread is passed under the artery with the help of a curved clamp, or a similar surgical tool. The thread is cut into two portions and moved in opposite directions: one towards the head, the other towards the thorax. On each portion, both tips of the thread are clamped and secured with a mosquito hemostat or similar. By pulling the cranial thread with one hand, the other hand is used to make a small incision (3–4 mm wide) in the exposed surface of the carotid—deep enough to expose the lumen (the inside) of the artery (some serum may ooze out, Figures 2A,B).

### Insertion of the cannula (cannulation of the vessels)

After opening the carotid wall, the next step is to insert a cannula into the lumen of the carotid artery. The cannula needs to be of sufficient gauge to allow the passage of a large volume of fluid at an appropriate speed to flush the blood and pass the fixatives big enough to allow easy flow. Although a variety of cannulas can be used, we routinely use a laboratory 2 ml plastic pipette whose outside diameter (5 mm) is a little smaller than the average lumen of a postmortem carotid artery. The tip of the pipette is conical in shape and blunted, so it does not tear the endothelium (which would be an obstacle to the passage of the fluid flow). After insertion, the pipettes are strongly tied to the wall of the carotid artery with the threads to prevent reflux. We use the following procedure: take the upper thread (closest to the head) and tie a tight knot to the wall of the vessel with the pipette inside and secure it by two or three additional knots. Then, take the lower end of the thread and do the same but passing the thread behind the pipette, holding the deep part of the artery, and again tying it with a tight knot. It should be noted that the endothelial surface of the artery is very smooth and slippery, so it is always necessary to tighten the ligatures to the carotid artery as much as possible (Figure 2B). The procedure is repeated on the other side of the neck with the same approach.

### Perfusion fluids

A critical step is the exit of blood and other perfusion fixatives out of the vascular system of the brain, to avoid damage by excessive pressure. In our protocol, the right side of the neck is chosen to continue the dissection of the carotid sheath and identify the jugular vein. Care must be taken due to the weak wall of the veins, with the risk of heavy bleeding and obscuring the dissection field if a tear in the vein occurs. Once the jugular vein is identified and freed from surrounding tissue, it can be either sectioned directly, or, if possible, two threads are passed under the vein (one closer to the brain and the other closer to the heart). The jugular vein is then pulled and sectioned to allow the drainage of the perfusion fixatives. The process should not take more than 10–20 min per side, adding about 30–45 min to the PMI. The time required to cannulate both carotids is variable, according to the difficulty in gaining access to them, as this depends largely on the anthropological characteristics of the body. For example, if a large amount of fat is present in the neck, this increases the time necessary to locate the carotids, and usually, the bleeding is heavier than in a lean person. Likewise, the bleeding of intervening veins in the neck can delay the whole procedure. Once the cannulae are well secured to the carotid wall, the process of passing the perfusion fluids starts.

### Blood drainage of the brain

This step aims to flush out the blood of the brain, which is drained through the sectioned jugular vein (see Section “Discussion”). The solutions flow cranially into the brain through the internal and external carotids (fascial tissue may also become fixed). The cannulae already inserted into both carotids are linked to a peristaltic pump whose flow can be adjusted (Figure 2C). One end of the peristaltic pump tube, previously flushed with saline to remove air bubbles, is introduced into the flask with saline. The other end is directed to both cannulae inserted into the carotid arteries through a “Y” connector.

The volume perfused by the peristaltic pump is adjusted to a flow of 200 ml/min, for 20 min for blood removal (i.e., 4,000 ml in total). This flow rate produces a pressure high enough to overcome small clots and partial obstruction of the cerebral vessels, but moderately to avoid bursting blood vessels (the suitability of these parameters has been demonstrated through a great deal of experience in nonhuman primates). The first four liters of fixative are passed at the same rate, and thereafter, the flow rate is lowered to 100 ml/min for the remaining 4 L of fixative (8 L in 60 min). The speed and volume should be titrated and adjusted to the specific pump settings, as these vary according to the model, brand, and elasticity of the connecting tubing.

### Solutions and perfusion sequence

#### Flush of the blood from the vessels in the brain

The first solution infused is 4 L of saline (0.9% NaCl) in 0.1 M phosphate buffer, at a pH of 7.4. In our protocol, the solution is prepared in advance and maintained at 4^°^C until use. The flask containing the saline is placed on a stirring plate and 50,000 U of sodic heparin are added to the saline just before the beginning of the perfusion (two 5 ml vials each containing 25,000 U of heparin). At this point, the peristaltic pump can be turned on to begin flushing the fluids through the brain. This procedure ends when the 4 L of saline has already passed. Immediately before the pump is started, the jugular vein on the right side is cut to allow the blood to drain freely. Obviously, a large amount of blood follows the section of the jugular vein, but once the carotids are cannulated, it is no longer a problem, and the mixture of blood and saline falls into the collecting system.

#### Fixation of the brain

A total of 8 L of 4% paraformaldehyde are passed through both carotids simultaneously. The first 4 L are passed at a rate of 200 ml/min, for about 20 min (same rate as the saline flushing), while the second 4 L are introduced into the vascular system at a lower rate, 100 ml/min, for another 40 min. The total time for intravascular perfusion is 1 h. We use two 4 L flasks and place 2 L of 0.2 M phosphate buffer at pH 7.4 (prepared in advance) into each flask. The phosphate buffer concentration is double than usually used, so it results in a final concentration of 0.1 M when mixed with paraformaldehyde. A stock solution of 8% paraformaldehyde in distilled water (twice the final concentration) is prepared and stored in dark bottles at 4^°^C until use. Now, 2 L of 0.2 M phosphate buffer is mixed with 2 L of 8% paraformaldehyde, yielding a total volume of 4 L of 4% paraformaldehyde per flask. For convenience, as mentioned, we use two flasks, each with 4 L of the fixative, so it is only the speed of the peristaltic pump that needs to be adjusted. Fixative solutions prepared in this way are effective for several weeks and are only mixed in the prosection room, immediately before beginning the exposure of the vascular system of the brain.

#### Fixation of the body

The intracarotid perfusion of the brain is complete after the 8 L of paraformaldehyde has been passed. The quality of the perfusion can be checked directly, as the face should be pale and hard due to the flushing of the fixative through the external carotid artery, from which the facial artery arises, thus fixing all tissues in the head.

**Figure 3:**
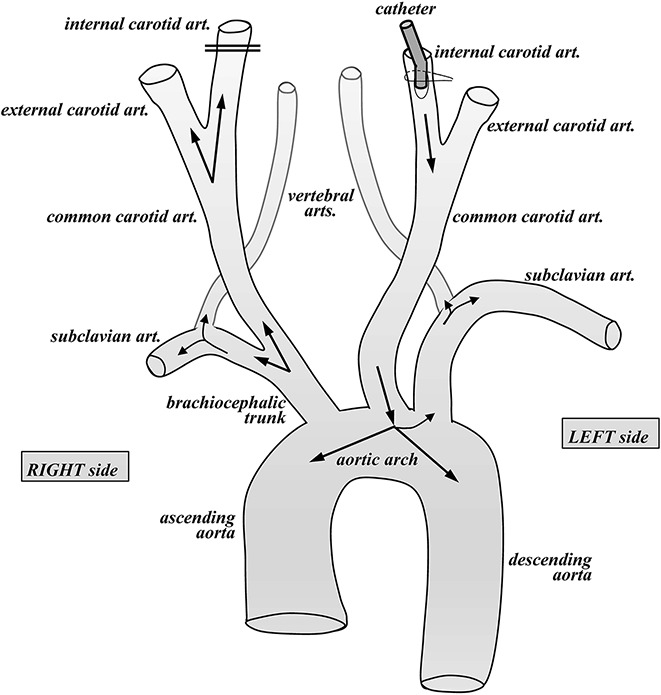
Diagram of the vascular organization at the level of the aortic arch with branches to the head and upper limbs. The cannula (catheter) is oriented to the aortic arch for fixation of the donor body; the vessels to the brain are obliterated.

As the body donation program is intended for educational purposes for various health science schools, we carry out the preservation of the body after brain intracarotid perfusion. We use this procedure to make maximal use of the body donation program. However, in this case, the fixative is changed to 10% buffered formalin (Panreac, Spain), which is another fixative widely used in human brain studies. We prevent the contact of the body fixative with the brain as much as possible, since it is mixed with an antifungal agent (0.5% Antimonium oxide III, Panreac, Spain) immediately before the perfusion of the body, and thus is not very compatible with histological processing of the brain. To this end, we first remove both cannulae from the carotids. On the right side, the upper and lower threads used previously to secure the cannula are tightly tied on the carotid wall, to close it completely, and prevent access of the body fixative into the brain. Once the right carotid has been removed, the left carotid is used to perfuse the body, which is started with the removal of the left carotid cannula. At this point, the carotid opening is then slightly enlarged with scissors to insert another different, wider cannula, with the tip directed towards the heart. The part of the carotid closer to the head is tied tightly, while the body perfusion cannula is inserted in opposite direction, that is, towards the heart, and secured to the left carotid. In this way, the flow of the fixative solution is directed towards the aortic arch, through which the body can be fixed completely. With this approach, we avoid passing 10% formalin with the antifungal agent into the brain through the carotids (Figure 3), although a small amount of the body fixative may reach the brain through the vertebral arteries.

#### Extraction of the brain

Once the fixatives have been passed completely, the brain is left *in situ* for 24–48 h, to allow the complete fixation of the body by diffusion of the formalin. This body fixation shows firmness of the muscular masses and skin in the trunk and limbs. Nonetheless, if required, the brain can be extracted immediately after the end of the intravascular fixation of the brain, in which case the body can be returned to the family for burial.

The brain extraction starts with changing the position of the body, which is moved to another table (that can be adjusted in height) and is placed in the supine position. The approach to remove the skull is the routine autopsy procedure, with some modifications. The main difference between the removal of a fixed and unfixed brain is the rigidity in the case of the fixed brain, which means special care is needed to retrieve the brain without damage to the cortical surface.

**Figure 4:**
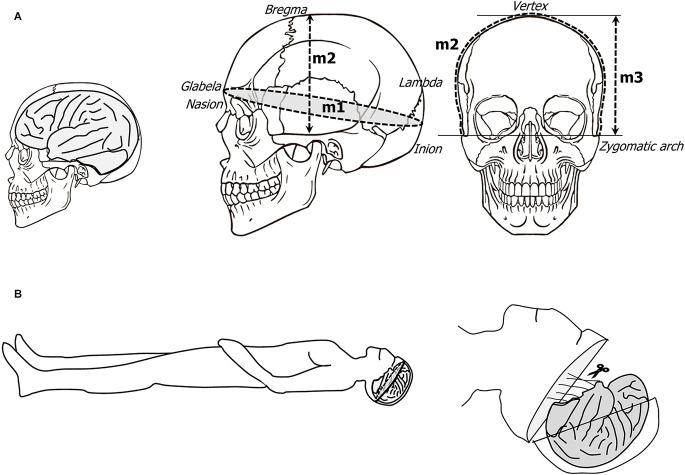
**(A)** Schematic representation of the craniometric points with the cranial measurements. **(B)** The body is placed in a supine position, and the skull is delineated from frontal to occipital bone through temporal bone; the skull is carefully cut and the brain is progressively removed after gradual sectioning of the cranial nerves from frontal to occipital region ending with the spinal cord.

First, the length, breadth, and height of the cranium are measured as a surrogate for intracranial volume, as this is not available from imaging (Figure 4A). The skin is incised along the interauricular line and both frontal and occipital flaps are reflected in opposite directions, so the skull is free of attached muscles (frontal and in particular the nuchal musculature).

**Figure 5:**
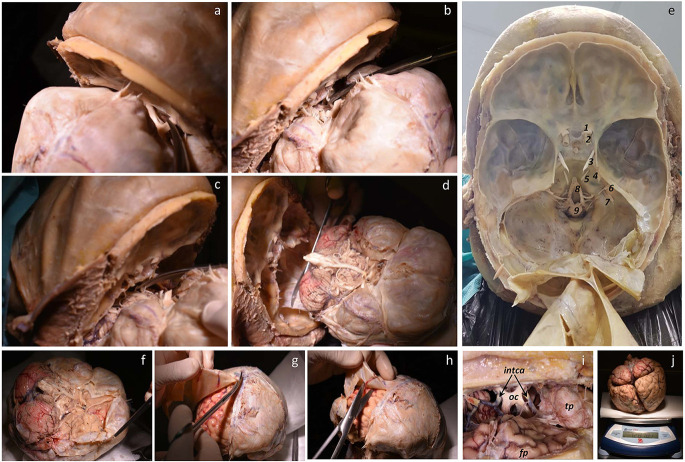
**(A–D)** Sequential sectioning of vessels, cranial nerves, and medulla. **(E)** Cranial base showing the anterior, middle, and posterior fossae covered by the dura mater, and tentorium reclined. Right blood vessels, cranial nerves, and medulla are shown (1. optic nerve, 2. carotid artery, 3. oculomotor nerve, 4. maxillar nerve, 5. abducens nerve, 6. facial and vestibulocochlear nerves, 7. glossopharyngeal, vagus, and accessory nerves, 8. vertebral artery participating to form the basilar artery, 9. medulla). **(F–H)** Base, superior, and lateral views of the removed brain showing a step for removing the dura mater. **(I)** Detail of the optic chiasm (oc), internal carotid artery (intca), frontal pole (fp), and temporal pole (tp). **(J)** Finally, the brain is weighed.

The skull is opened with an oscillating saw in a circular fashion around the perimeter of the head, with the line of opening as low as possible. The frontal part of the opening lies close to, or even enters the cavity of the frontal sinus. The opening is continued caudally through the temporal bone; before this step, a detachment of the temporal muscle on the external aspect of the temporal bone (superior temporal line) is needed to facilitate a line of bone section as low as possible. The occipital line of opening also needs to be performed as low as possible, which requires the previous detachment of the musculature (trapezius, splenius capitis and semispinalis capitis muscles that insert in the superior nuchal line) to leave the occipital bone under the superior nuchal line free. Since the circular line of bone opening is now complete, the calvarium can be detached from the surface of the dura mater (Figure 4B). The next step is the opening of the dura mater. We start detaching the boundary of the dura mater and the process of the frontal bone known as *crista galli*. The dura is cut longitudinally with scissors along a line 0.5 cm away from the midline, parallel to the superior sagittal sinus, as far as the confluence of the superior longitudinal and transverse sinuses (torcula). The cutting of the dura is continued in a lateral direction, and the flap is reflected towards the back of the head. The attachment of the anterior tip of the dura mater to the *crista galli* process is now sectioned, and the midline dura mater (*falx cerebri*) is gently retracted backwards. The surface of the frontal lobe can be retracted backwards very gently, thus exposing the olfactory bulbs and olfactory nerves. Continuing to gently push the frontal lobe upwards, both optic nerves become visible at the bottom of the anterior cranial fossa, and they are also sectioned. The entry of the internal carotids can be identified immediately behind the optic nerves, and they are also cut. A difficulty arises at this point, as the temporal pole cortex needs to be retrieved as intact as possible. The temporal pole is the most anterior portion of the temporal lobe, and it lies under the free edge of the lesser wing of the sphenoid bone. The shape and depth of the middle cranial fossa varies from case to case; therefore, it is necessary to proceed with great care. However, in some cases, some tear of the temporal cortex cannot be avoided, though it should at least be minimized. Once the temporal poles are liberated and the optic nerves are sectioned, the ventral surface of the hypothalamus becomes visible, and with it, the dural diaphragm of the *sella turcica*. We leave the hypophysis in place, but if its retrieval is necessary, this can be achieved by cutting the dura mater that covers the hypophysis. The oculomotor cranial nerves III, IV, and VI, and the ophthalmic branch of the V cranial nerve are visible towards the lower orbital fissure. All these structures are cut with scissors (Figure 4B). By retracting the brain, the anterior portion of the mesencephalon is visible. Next, the dorsal surface of the tentorium is exposed, which needs to be sectioned to allow the retrieval of the cerebellar hemispheres. Beginning at the anterior portion, the tentorium is cut laterally, thus exposing the dorsal surface of the cerebellar hemispheres. By gentle retraction of the brain, the ventral surface of the brainstem is first visible, followed by the remainder of the cranial nerves, which can be cut at this time: V, VI, VII–VIII, IX–X, XI–XII (Figures 5A–C). Finally, the medulla is sectioned at the bulbospinal transition, after which, the brain is totally retrieved (Figures 5D,E).

#### Examination of the brain

The following steps must be taken in a hood with appropriate ventilation and fume extraction. The brain is positioned such that its ventral surface is exposed, and the vasculature is visible at the base of the brain. A close and systematic examination of both the vasculature and external surface is carried out to take note of the presence of pathology, in particular atherosclerotic plaques. First, we examine both carotids and the first segments of the middle cerebral artery, looking for the size of the vessels, the presence of plaques, and the plaque density. We then proceed to examine the anterior communicating artery and the beginning of the anterior cerebral arteries following the same routine. Next, we examine the posterior communicating and anterior choroidal arteries and finish with the posterior cerebral, basilar, and vertebral arteries, taking note of any plaques in the vessels. This examination provides information on the state of cerebral vessels, so in case of lack of fixation of a vascular territory, the artery responsible is checked. In this way, we evaluate the outcome of the intracarotid perfusion.

#### Postfixation

At this point, the dura mater has been totally removed. The brain is weighed on a scale and placed in a wide container with a hermetic cover to avoid evaporation and formalin fumes, with the ventral surface up, and abundant gauze at the bottom (Figures 5F–J); 3 L of freshly prepared 4% paraformaldehyde is poured into the container, or enough to cover the brain. On top of the brain, we place additional gauze soaked in 4% paraformaldehyde. The brain is then stored in a cold room (4^°^C) for postfixation, where it remains intact until processing for study. The time of postfixation is variable, according to the research needs, but we have processed brain tissue up to 5 years after intracarotid perfusion, both at the microscopic and ultrastructural levels with good results (see below).

#### Removal of the superficial blood vessels, photography, and brain blocking

The last step is the preparation of the brain for photographic records and a more permanent deposit until histological use. The first step is the removal of the pia mater and superficial brain vessels. Additional caution needs to be taken considering the fragility and lack of flexibility of the fixed brain at this step. First, we start with the biggest vessels of the circle of Willis (basal surface of the brain) by cutting the communicating arteries with fine scissors. Gently, the carotid and middle cerebral arteries are pulled along the pia mater that covers the lateral fissure, the arterial and venous branches coming off easily. This process is continued from the lateral fissure towards the midline of the brain, thus removing the frontal and parietal regions of the hemisphere. The same procedure is employed moving towards the temporal lobe, although the vessels are not as big on the lateral surface of the temporal lobes. Blood vessels in the occipital lobe are smaller in size and tend to stick to the surface of the brain, so this requires great care. It is not advisable to remove every little vessel on the brain surface; we leave small vessels, mostly veins that still contain a little blood but do not impair fixation.

We repeat the procedure on the other hemisphere, and then place the brain with the ventral surface up. The small arteries of the temporal pole are removed, after which the anterior choroidal and posterior communicating arteries are identified and pulled away with forceps, taking care not to damage the medial temporal lobe regions such as the hippocampus or parahippocampal gyrus. The vessels should be pulled perpendicularly to the surface of the brain, to avoid oblique tractions that may result in tearing the cortical surface. Other small branches of the posterior cerebral artery, such as the calcarine branch are the last vessels removed. Once the lateral and medial surfaces of the brain are clean of vessels and pia mater, we continue with the medial surface of the brain. We take the pia mater from the lateral aspect of the frontal and parietal lobes and, after identification of the anterior cerebral artery and its largest branches (the callosomarginal and pericallosal arteries), all are removed on the medial side of the hemisphere. The removal of the pia mater in this brain region is usually incomplete, although it can be completed once the hemispheres have been separated (see details below). The brainstem is also cleaned by the removal of the pia mater and vessels. The superior cerebellar artery is identified and removed from the dorsal (upper) part of the cerebellum. After this, the basilar artery and its largest branch, the inferior anterior cerebellar artery, along with the finer pontine branches, are removed followed by the inferior posterior cerebellar artery over the inferior olive, and the vertebral arteries. At this point, the removal of pia mater and vessels is largely completed. The brain, now freed from pia and vessels, is ready to be photographed to record the morphology of sulci.

**Figure 6:**
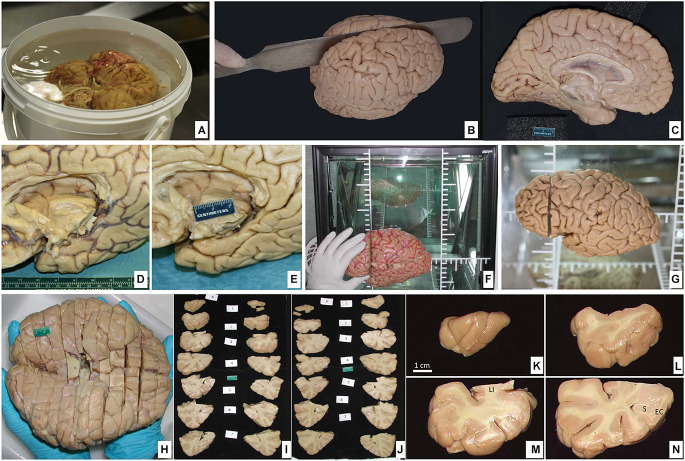
**(A)** Postfixation of the brain maintaining the ventral surface resting on the bottom of the container protected with surgical gauze. **(B)** Dorsolateral view of the brain being cut through the midline separating the hemispheres. **(C)** Medial view of the left hemisphere. **(D)** Ventromedial side of the brain showing the callosal corpus and the medial temporal lobe; blood vessels are disposed on the sulcus. **(E)** Large vessels along with the pia mater are removed. **(F)** The brain hemisphere is placed in a podoscope, matching the horizontal line with the intercommisural line (ac-pc); thus, a first cut is made perpendicular to the ac-pc line, at the anterior commissure level. **(G)** The first cut is shown. **(H)** Ventral view of the full brain, sectioned into slabs (roughly 1 cm-thick). **(I,J)** Both anterior and posterior views of the slabs are sequentially photographed for each of the two hemispheres. **(K–N)** The four first slabs of the temporal lobe in the left hemisphere are shown in detail at the anterior face (LI, *limen insulae*; S, subiculum; EC, entorhinal cortex).

Once photographed the outer surface of the complete brain in various projections, it is first necessary to separate the brainstem from the remainder of the brain. This step is performed by sectioning the mesencephalon. Looking at the ventral side of the brain, the cerebral peduncles and the interpeduncular fossa are exposed facing the anterior part of the brain. Both cerebral peduncles are cut, and the section is extended towards the dorsal part of the mesencephalon at the level of the superior colliculus. The cerebral hemispheres are separated from the brainstem (Figures 6A–C). In the second step, the brain is bisected. To do so, we place the brain on its ventral surface and begin with a cut that passes through the middle of the optic chiasm and continues through the midline of the infundibulum of the hypothalamus, continuing through the narrow space between mammillary bodies, thus entering the cavity of the third ventricle. The section is continued as far as the complete section of the splenium of the corpus callosum. At this point, the fornix on both sides, linked posteriorly to the contralateral fornix by the hippocampal commissure or psalterium (macroscopic)^2^, is sectioned. The brain hemispheres lie wide open, exposing the inner surface of the third ventricle, the interventricular foramen of Monro, and the upper part of the lateral ventricle. The section follows the midline marked by both fornices as far as the anterior commissure that is also cut, fully separating the two hemispheres. This completes the bisection of the brain, resulting in three independent pieces (two cerebral hemispheres and one brainstem) available for photographic recording. We take photographs of all pieces from at least four projections: dorsal, ventral, medial, and lateral surfaces. The photographic record is invaluable for the interpretation of the brain slabs and their spatial continuity (Figures 6D,E).

**Figure 7:**
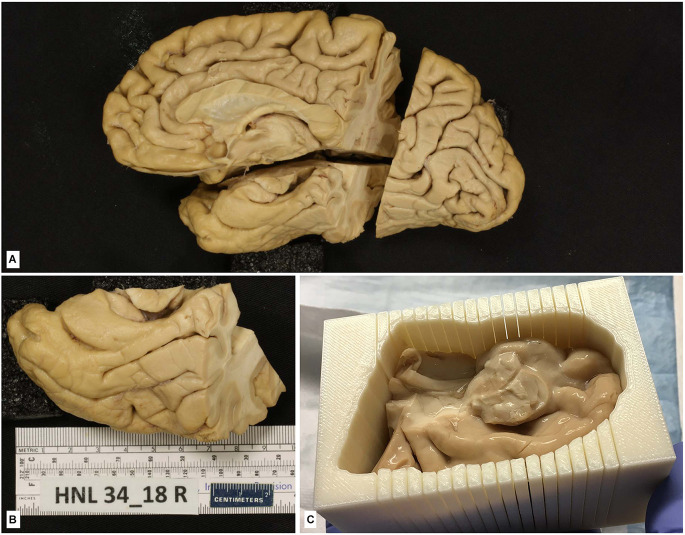
**(A)** The hemisphere of one of the cases of the series in Yushkevich et al. (2021). The brain hemisphere is divided into three blocks by a perpendicular cut to the *ac-pc* line extending into the temporal lobe, which is further isolated for that particular study. **(B)** Isolated temporal lobe piece. **(C)** Temporal lobe is embedded in a mold built following a process of 3D reconstruction of 7T MRI images of the temporal lobe using a 3D printer; the mold shows parallel slits in the same plane as MRI images separated by 5 mm; the plane of section of the temporal lobe is kept as following slits at 2 cm.

Blocking of the brain varies according to specific needs. What follows is the method we use for the storage of brain tissue in slabs, as outlined in Insausti et al. (1995). The plane of the section we choose corresponds to the line perpendicular to the anterior-posterior commissures (Mai et al., 2015). To achieve this, we place one hemisphere on its medial surface on top of a glass at a distance of 2–3 cm from a mirror that reflects the image of the medial side. The glass has etched lines perpendicular to one another; with this guide, the anterior and posterior commissures are aligned, and with a brain knife, a first cut is made through the whole hemisphere (Insausti et al., 1995). While this first cut can be made at any point along the bicommissural line, we take the anterior commissure as a reliable and reproducible point that can be easily identified; a cut at this level leaves a rostral block, from the anterior commissure to the frontal pole, and a caudal block, from the anterior commissure to the occipital pole (Figures 6F–H). The resulting cut coronal surface is placed on a flat surface; two rods (1 cm thick) are placed along the medial and lateral aspects of the block. The brain knife, supported by the medial and lateral rods, is passed through the frontal block of the brain, gliding the knife, on top of the rods, and parallel to the first coronal cut. The resulting slabs are set apart for further photographic recording (Figures 6I–N). The process is repeated until the frontal cortex tip is reached. The same procedure is followed on the caudal block as far as the occipital lobe. An example of the procedure can be found in Insausti et al. (1995). Different studies may require more intact brains. These can range from the whole brain (Annese et al., 2014), one hemisphere (Sadaghiani et al., 2022), or a part of the hemisphere (Ravikumar et al., 2021; Yushkevich et al., 2021; Figure 7).

No specifications for general Nissl stain, histochemical, immunohistochemical, or confocal microscopy visualization are provided here, since detailed protocols can be found in the references in which brain tissue fixed with intravascular fixation has been used. For studies in which intact temporal lobe specimens are imaged with 9.4T MRI, the MRI procedures are described in Yushkevich et al. (2021). As described in this reference, MRI can be used to 3D print a custom mold that can precisely guide the sectioning of the temporal lobe tissue (Figure 7) and reduce the problem of image registration between the MRI volume and serially acquired histological images. Likewise, specific procedures for intracellular filling and electron microscopy are described in the “Results” section.

## Results

The protocol described above allows a faster fixation of the brain (compared to protocols reported in other studies), thereby minimizing the degradation of the nervous tissue. The success of the fixation is gauged by considering the results obtained in different studies with different techniques. Our line of work is focused on the hippocampal formation and the limbic system in general. Therefore, the examples provided here, from our and others’ laboratories, mainly refer to these brain regions.

### Serial histological processing

**Figure 8:**
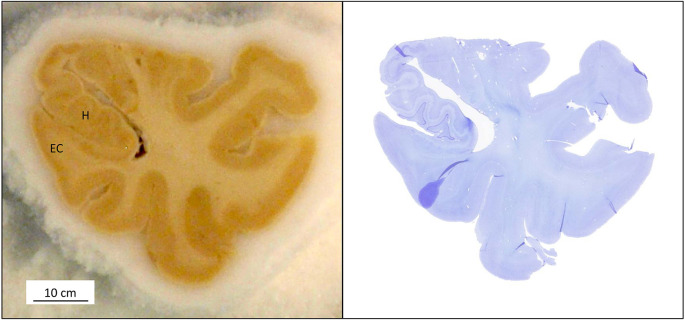
(Left) A sectioned block of the temporal lobe is positioned on the microtome stage with the medial side of the hippocampus (H); EC: entorhinal cortex. (Right) The Nissl and MRI correspondence are shown at the same level.

The quality of the fixation can be appreciated in the demonstrated structural preservation of the cortex, amygdala, and hippocampus, which are the focus of our research (Figure 8). The technique described above allows serial sectioning of large pieces of fixed brain (up to 8 cm long; Yushkevich et al., 2021). In these cases, the firmness of the brain, derived from the quality of the perfusion, made collecting all frozen sections in a straightforward way. Typically, we get serial sections at 50 μm, in a series of 10 sections from 2 cm slabs of the whole human temporal lobe in a sliding microtome coupled to a freezing unit. For each slab, we systematically obtain 40 complete series as well as some additional sections (less than eight sections). The seriation is repeated along all blocks, with a gap of 500 μm or less between adjacent blocks (Figure 8). From a single block—i.e., a block 2 cm in height sectioned at 50 μm thickness—40 series of 10 sections can be obtained (40 × 10 × 50 μm equals 20,000 μm, or 2 cm). We use series at an interval of 500 μm for serial reconstructions of the human temporal lobe after obtaining serial block-face photographs (Yushkevich et al., 2021). Regarding applicability to quantitative microscopy (stereology), the technique of carotid perfusion assures consistent histology across subjects that warrant the numerical estimation of multiple parameters (Artacho-Perula and Insausti, 2007; Delgado-Gonzalez et al., 2020).

### Myelin and histochemical procedures

**Figure 9:**
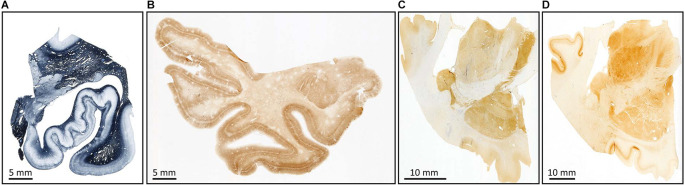
Examples of myelin stain with the Heidenhain method **(A)**; immunostaining for non-phosphorylate neurofilaments SMI-32 **(B)**, parvalbumin **(C)**, and calbindin **(D)**.

Brain sections from tissue fixed through the carotids were used in myelin staining by the method of hematoxylin (Heidenhain stain, as modified by Hutchins and Weber (1983; Figure 9A). Histochemical reaction of acetylcholinesterase by the method of Hedreen and Bacon (Hedreen et al., 1985) and the NADPH-diaphorase technique have also been processed and reported previously in other publications (De Lacalle et al., 1994; Sobreviela and Mufson, 1995).

### Immunohistochemical procedures

The goal of a brain fixation method is to facilitate the rapid entry of aldehydes into the brain, preserving epitopes, structure, and immunoreactivity (Cuello, 1993).

**Figure 10:**
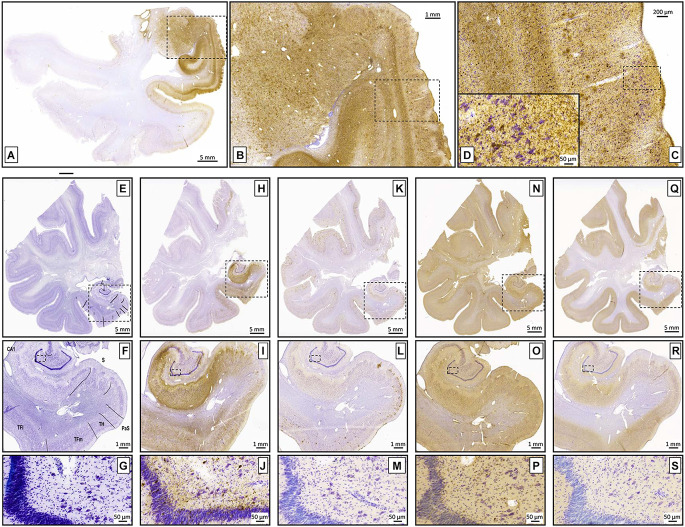
**(A–D)** Tau protein immunostaining. An example at different levels of magnification (dotted lines represent magnified zones). The inset in **(D)** shows neurofibrillary tangles and threads. The images show mild/moderate stage of Alzheimer’s disease. **(E–S)** Examples of different immunohistochemical markers in adjacent sections. **(E–G)** Nissl stain with annotations on the medial temporal lobe, followed by Tau **(H–J)**, β-amyloid **(K–M)**, TDP-43 **(N–P)**, and α-synuclein **(Q–S)**.

**Figure 11:**
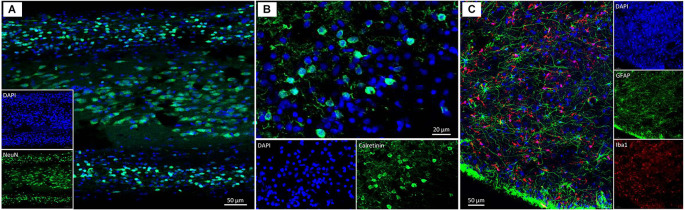
Confocal images of the human olfactory peduncle after the fixation process reported in this study. **(A)** DAPI and NeuN are merged. **(B)** DAPI and calretinin are merged. **(C)** DAPI, GFAP, and Iba1 are merged.

In the present study, we used a variety of antibodies in tissue fixed by intracarotid brain fixation, published previously. The antibodies that have proved useful over the years are included below, along with the articles in which they were reported. It should be made clear that the series of immunoreactions do not intend to be an exhaustive list. It has proven useful in:

(a) Structural cytoskeleton of neurons (non-phosphorylated neurofilaments SMI-32; Blaizot et al., 2010; Figure 9B).
(b) Calcium-binding proteins (Grateron et al., 2003; Figures 9C,D).
(c) Pathological markers such as Tau protein AT8 (Figures 10A–D). Consecutive sections: Nissl stain (Figures 10E–G), Tau protein (Figures 10H–J), β-amyloid (Figures 10K–M), TDP-43 (Figures 10N–P), and α-synuclein (Figures 10Q–S) (Ravikumar et al., 2021; Wisse et al., 2021b; Yushkevich et al., 2021).
(d) anti-NeuN (Tapia-Gonzalez et al., 2020; Dominguez-Alvaro et al., 2021; Figure 11).
(e) Peptides such as somatostatin-28 (Amaral et al., 1988).
(f) Myelin basic protein (MBP; Raspeño-Garcia et al., 2021).
(g) Glial markers (GFAP and Iba1; Raspeño-Garcia et al., 2021; Figure 11).
(h) Amino acids and transmitters (GABA, Serotonin, Tyroxine-hydroxylase, choline acetyltransferase; De Lacalle et al., 1994).

In all these immunoreactions, the quality of immunohistochemical detail was at least equal to the best immunoreactivity observed in samples from immersion fixed brains.

### Fluorescence and confocal microscopy

Double labeling is nowadays feasible with confocal microscopy, and the use of fluorophores of different colors and affinities is the most appropriate choice for multiple labeling. Our protocol of brain perfusion is perfectly compatible with the processing of human brain tissue and examination with confocal microscopy. We tried antibodies to demonstrate glial markers (Iba2, GFAP), as well as myelin basic protein (Figure 11).

### Intracellular filling

The work of Benavides-Piccione et al. (2020) shows how this technique of intracarotid fixation preserves the morphology of hippocampal neurons compared to other species such as mouse. The study was carried out in two cases. The PMI was short (3–5 h of PMI). After extraction, the brain tissue was fixed in cold 4% paraformaldehyde in 0.1 M phosphate buffer (pH 7.4 for 24–48 h). After fixation, the tissue was washed in phosphate buffer and sectioned coronally in a vibratome at 300 μm thickness.

**Figure 12:**
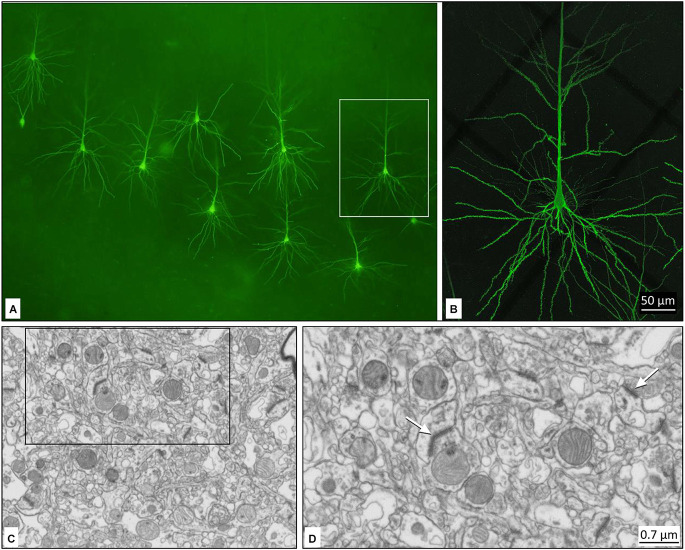
Confocal microscopy image of neurons injected with Lucifer Yellow in the human hippocampus. Low magnification image of labeled pyramidal cells in the CA1 field. **(B)** Higher magnification image of the boxed region in **(A)**, showing an injected CA1 pyramidal cell. For details, see Benavides-Piccione et al. (2020). **(C)** Low magnification image from layer III of the human temporal cortex showing the ultrastructure of the neuropil. **(D)** Higher magnification of **(C)**. Arrows indicate synapses. For details, see Cano-Astorga et al. (2021).

The resulting quality of the intracellular filling with 8% Lucifer Yellow in Tris buffer (pH 7.4) was excellent. The demonstration of the dye was prepared with rabbit antiserum at a dilution of 1:400,000 (generated at the Cajal Institute, Spain), diluted in a solution of 2% bovine serum albumin, 1% Triton and 5% sucrose. Finally, it was visualized with 1:100 biotinylated donkey anti-rabbit antiserum and streptavidin-conjugated Alexa-fluor 488 (1:1,000). Details of the procedure can be found in Benavides-Piccione et al. (2013) and Benavides-Piccione et al. (2020). The quality of the preservation of the neuronal morphology is revealed in the exquisite detail of dendritic arborization, including dendritic spines, with detail comparable to that obtained in mice (Figures 12A,B).

### Electron microscopy

Only optimal fixation allows preservation of the tissue ultrastructure, which is necessary for successful electron microscopic examination. Examples are shown in Figures 12C,D. Tissue from cases fixed through intracarotid perfusion has been used and reveals its capacity to preserve the ultrastructure. After extraction, brain tissue was fixed in cold 4% paraformaldehyde (Sigma-Aldrich, St Louis, MO, USA) in 0.1 M sodium phosphate buffer (pH 7.4) for 24–48 h. After fixation, the tissue was washed in phosphate buffer and sectioned coronally in a vibratome (150 μm thickness).

Sections containing the temporal cortex were selected and postfixed for 24 h in a solution of 2% paraformaldehyde, 2.5% glutaraldehyde, and 0.003% CaCl_2_ in sodium cacodylate buffer (0.1 M). The sections were treated with 1% OsO_4_, 0.1% potassium ferrocyanide, and 0.003% CaCl_2_ in sodium cacodylate buffer (0.1 M) for 1 h at room temperature. They were then stained with 1% uranyl acetate, dehydrated, and flat-embedded in Araldite for 48 h at 60^°^C (DeFelipe and Fairen, 1993). The embedded sections were then glued onto a blank Araldite block. Finally, three-dimensional electron microscopy was prepared (Figures 12C,D). Further details can be found in Cano-Astorga et al. (2021).

### Application to neuroimaging

**Figure 13:**
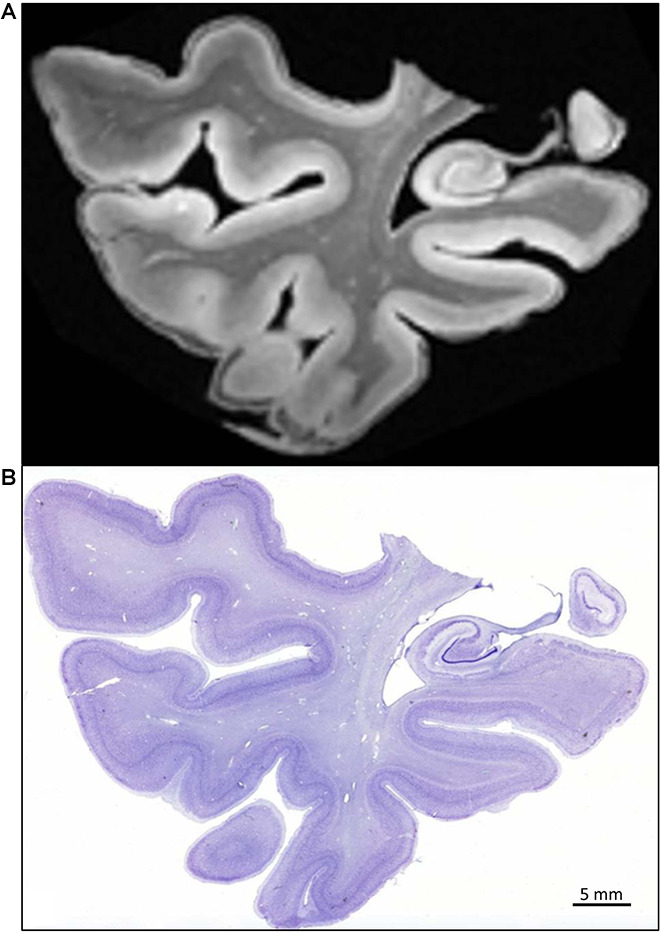
Images of one case of the series, showing the correspondence of a level at 9.4T MRI image [upper panel, **(A)**], and the adjacent Nissl stain **(B)**. Notice that the level corresponds to the *Gyrus intralimbicus*, at the end of the uncus.

Brains fixed with our protocol of intracarotid perfusion have proven useful for obtaining *ex vivo*, high-resolution images (at 9.4T and 7T) in studies of the human temporal lobe. Morphological properties, such as volumes and thickness of the medial temporal cortex subregions and hippocampal subfields could be obtained readily using manual and automatic approaches, in turn allowing quantitative analyses that correlate brain structure with the burden of proteinopathies in neurodegenerative diseases (Ravikumar et al., 2021; Wisse et al., 2021b). Well-preserved tissue facilitated MRI procedures, including the use of MRI to guide serial histological sectioning described in Section “Immunohistochemical procedures” above. The use of MRI to create a custom 3D-printed mold, and the use of this mold to guide the sectioning of histology simplified the problem of MRI-histology registration resulting in registration errors in the order of a single MRI voxel (Yushkevich et al., 2021). This in turn made it possible for anatomical boundaries to be mapped from histological slides to 3D MRI space, for the first time generating sequential 3D segmentations of the medial temporal lobe that are based on cytoarchitecture, rather than MRI contrast and geometrical rules (Ravikumar et al., 2021). It also made it possible to generate first-of-their-kind 3D maps of the p-tau neurofibrillary tangle burden in MRI space (Yushkevich et al., 2021). Maintaining the plane of the section allows accurate matching of *ex vivo* MRI and Nissl-stained or immunohistochemical series (e.g., protein Tau), as reported in Yushkevich et al. (2021) and Ravikumar et al. (2021) (Figure 13).

### Comparison of *ex vivo*, *ex situ* with *ex vivo*, *in situ* vascular perfusion of the brain

Both procedures have been demonstrated to be equal in histological and immunohistological studies (e.g., Amaral et al., 1988; Insausti et al., 1995). No other techniques (such as intracellular injections, confocal microscopy, or electron microscopy) were tested, and no conclusions can be drawn on possible differences between immersion or perfusion methods for human brain fixation. However, some deformation of the brain was noted after the fixation because of the weight of the brain itself (from being placed in ventral or lateral positions to allow visualization of the brain vessels); this could be troublesome in MRI-histology studies.

## Discussion

Several different protocols have been proposed for the optimal preservation of the human brain structure for morphological studies. Our method of vascular fixation through both carotid arteries at the same time differs from that employed in other studies (Waldvogel et al., 2006; Alkemade et al., 2020; Maranzano et al., 2020) with the vascular approach being the only point in common. While all these studies involved perfusing through the vascular system, none of the studies to date have been designed to take advantage of an anatomical setting; in addition, the protocols for perfusion are not provided, unlike the present study, in which the procedure has been described in detail. We report a single, vascular fixation protocol which has proven reliable in several different histological and neuroradiological procedures. The method proposed here is *ex vivo*, *in situ* (postmortem, intracranial) fixation of the brain through both carotid arteries simultaneously. It has been demonstrated that vascular perfusion *ex vivo*, *ex situ* (postmortem, brain extracted) is the best approach for epitope preservation in immunohistochemical methods (Beach et al., 1987). The fixative starts acting more widespread and a more homogeneous distribution of the fixative is obtained by diffusing from the inside of the brain, and not just from the brain surface to the center of the brain and white matter, even when the brain is bisected, and the midline and lateral ventricle are exposed. However, most human brain morphological studies use immersion-fixed brains.

### Procurement of human brains

There are several variables to be considered when predicting possible drawbacks in the quality of the fixation of any brain. As described above, this method promotes cooperation with anatomy departments, allowing them to provide human brains, while simultaneously maintaining the human bodies for teaching purposes. Our method takes advantage of the cannulation of the left carotid to direct the fixative to the general circulation through the aorta, in a direction opposite to that used for brain fixation (McHanwell et al., 2008)^3^. Anatomy departments usually have human body donation programs, and these donations could be used to perfuse both the brain for research purposes and the body for anatomical studies. This constitutes a novel and simple approach to encourage brain fixation in body donors in an anatomical setting. It should be noted, however, that some limitations may hinder the wide use of our method since most neuroscience laboratories lack anatomical facilities or close contact with anatomy departments. Nevertheless, this protocol could be very useful to obtain human brain tissue if implemented in an appropriate setting such as an anatomical laboratory where embalming takes place.

### Postmortem interval

The PMI of our series is below the averages of other series reported, which are often more than 10 h (e.g., Bian et al., 2016; 24 h, Beaujoin et al., 2018; 36 h, Alkemade et al., 2022). In our series, the average of 7 h delay between death and the beginning of the perfusion was largely due to the time required for coordination with funeral directors. In this respect, the University of Castilla-La Mancha has an institutional agreement with a funeral company that recognizes the value of bringing the body to the anatomical facilities of the school of medicine.

### Brain fixation

The best approach to study, human brain tissue in a reliable and reproducible way is to make use of the vascular route to deliver fixatives into the brain. We have described how a short PMI—together with the flushing of the blood using saline with heparin letting the venous blood escape—allows the fixatives (freshly prepared 4% paraformaldehyde in 0.1 M phosphate buffer) to readily fix the brain. Paraformaldehyde is a polymer of formalin, and therefore corresponds to methanal, a small molecule of low molecular weight that crosses readily from capillaries to the neuropil in similar tissue conditions to those in experimental animals. Fixation via a perfusion system is a method that is available to most, if not all, anatomy departments worldwide. Such departments are in an optimal position to collect body donations for human brain research purposes, likewise, to teach the human body in medical and health sciences in general, and the practicing of surgical skills by a variety of specialists. Our laboratory (Human Neuroanatomy Laboratory) has been using this method for many years, supported by the procurement of human brains through our body donor program, with a short PMI for morphological studies of the human brain.

### Anatomical dissection skill requirements

The necessary equipment for performing the anatomical exposure of the carotid artery is presented in Table 1. The surgical material is basic and accessible to many surgical brands.

**Table 1:** Detailed list with all the equipment needed for *in situ* perfusion.

• Autopsy table (preferably with fumes aspiration) in a well-ventilated room. • Surgical material: scalpel, forceps, scissors, retractors, hemostats, curved surgical needles, thread, ….). • 2 ml plastic pipettes (5 mm OD, or better arterial cannulae. • Glassware appropriate for preparing and holding the solutions (flasks, stirring plate, ….). • Peristaltic pump. Any brand where the output can be controlled is useful, once it is titrated to the volume yielded. The pump is linked to a “Y” connector that splits the flow to both carotid arteries simultaneously.
Brain perfusion
• Washing solution: Perfusion of the brain with infusion (4 L; 0.9% NaCl) in 0.1 M phosphate buffer, at a pH of 7.4 (first solution). Add 50,000 U of sodic heparin (two 5 ml vials each containing 25,000 U of heparin). • Perfusion solution: 4% paraformaldehyde in 0.1 M phosphate buffer.). Perfusion rate: 200 ml/min for 20 min (i.e., 4,000 ml total). Afterwards, lower to 100cc/min for 40 min.
Body perfusion
• Body perfusion: 10% buffered formalin (Panreac^°ledR^, Spain) mixed with an antifungal agent (0.5% antimonium oxide III, Panreac^°ledR^, Spain).
Brain retrieval
• Scalpel, forceps, scissors, hammer, and wedge for separation of the cranium bone, oscillating saw. • Scale for the weight of the brain. • Container (3 L), with gauzes (4% paraformaldehyde). • Cold room (4^°^C; postfixation).

The method we propose requires some anatomical knowledge of neck anatomy. In case no technical help is available, most anatomical dissection atlases for medical students will be of use in the steps for exposing and cannulating the carotid arteries and sectioning the jugular vein. Likewise, a certain familiarity with brain vasculature and its anatomical variability is also needed (van Donkelaar et al., 2020). The method presented here shows difficulties inherent to practicing human cadaver dissection of the carotids and jugular veins in the neck. However, accomplishing the protocols described in the “Methods” Section should be feasible for anatomy demonstrators, medical doctors, medical students, mortuary technicians, etc. The superficial location of the structures to be dealt with and the surface anatomy of the reference points allow the vascular structures to be located. To the best of our knowledge, no other study describes the dissection protocol to access both carotid arteries in the neck for brain fixation in detail. Although our method does not involve cannulation of the vertebral arteries, this does not negatively affect the quality of the brain fixation (see below). It would be very difficult to meet the need of preserving the neck anatomy as much as possible if the two vertebral arteries were to be identified and cannulated, as a broader and deeper dissection of the base of the neck—or even the cutting off of the head—would be required (Alkemade et al., 2022).

Therefore, rather than a limitation of the method, we would suggest that this increases cooperation between laboratories and institutions and increases the awareness of the difficulties associated with studying the human brain.

### Quality of the perfusion

Once the brain is retrieved, the surface of the brain looks pale, although some small vessels (usually small veins that are firmly attached to the pial surface) can be observed. In our method, the posterior part of the brain (occipital lobes at the lateral and ventral surface) shows small cortical vessels containing blood. This region corresponds to the vascular territory of the posterior cerebral artery coming from the vertebro-basilar system, through the vertebral arteries. These are not cannulated, in contrast to the method used by Alkemade et al. (2020), in which cutting the head of the donor allows easy access to the four vessels that irrigate the brain. However, such a procedure precludes large series of cases. The fact that small vessels are not washed does not prevent a good fixation through the posterior communicating arteries, which link the carotid and vertebro-basilar systems. The flow of the flushing solution would travel retrogradely from the posterior communicating artery to the posterior cerebral arteries, and flowing retrogradely, to the basilar and vertebral arteries with their branches to complete the fixation of the brainstem. The firmness of the cerebellum and brainstem after extraction indicates an adequate fixation by our method. Furthermore, there is the trade-off between having a perfectly secured, four-vascular trunk perfusion and the inevitable lengthening of the PMI until the fixatives enter the brain vascular system. Therefore, our procedure represents a balance between the two approaches (two vessels vs. four vessels), with the advantage of our method outweighing the possible drawback. Moreover, our method always postfixes the brain, thereby compensating possible problems of intracarotid perfusion. We suggest that our method provides a homogeneous fixation of the brain (were this not the case, the under-perfused brain regions would show a pink appearance due to incomplete flushing of the brain). A measure of the perfusion effectiveness of our method is the absence of blood visible in the brain vessels over the cortical surface, in contrast to immersion fixation techniques, thereby reducing the step of elimination of endogenous peroxidase. Our method does not provide control over the pressure at which the fluids are introduced into the brain vasculature. Instead, we rely on our broad experience of perfusion of nonhuman primates (Insausti and Amaral, 2008; Mohedano-Moriano et al., 2015), in which we perfused the same fixative, at the same volume rate (flushing at 200 ml/min, fixation at 200 ml/min followed by 100 ml/min, but not a final wash with 5% sucrose), with optimal results in terms of brain fixation. We intend to further explore the control of volume and pressure rates in the fixation step, as well as other modifications of the fixation fluids (e.g., trying fixatives with glutaraldehyde).

### Brain extraction

As far as timing is concerned, brain extraction is the variable that can be adjusted to adapt to specific needs and circumstances once the brain has been perfused. The extraction of the brain can be delayed according to the time required for the fixation of the body. In one case, the delay between brain perfusion and brain extraction was extended up to 72 h, achieving Nissl-stained appearance, although this case was not used for other techniques.

Other than the time elapsed between fixation and retrieval, one of the major difficulties of removing the fixed brain from the skull is tearing or damaging the cortical surface of the brain when extracting it. The firmness of the brain contrasts with the retrieval of a brain which has not been fixed, in which case the softness and flexibility of the brain facilitate the extraction, after severing cranial nerves and vessels. Following *ex vivo, in situ* fixation, the brain is firm and hard, which makes it difficult to remove parts of the brain encased in rigid structures, namely two structures in particular: the temporal pole and the cerebellum with the brainstem. The temporal pole is enclosed (tightness is variable) by the lesser wing of the sphenoid bone. The retrieval is difficult and needs to be done very gently, while carefully checking other points of the anterior temporal gyri. It may also be necessary to remove with rongeurs part of the lesser wing of the sphenoid, as it forms a roof on the temporal pole. Another site where difficulties may be found is the extraction of the brainstem along its edge with the cerebellum. The cerebellum is covered by a dura mater fold the cerebellar tentorium that separates the occipital and ventral temporal lobes from the dorsal part of the cerebellum. In this step of the brain extraction, the tentorium must be cut as far laterally as possible, so that the brain with the brainstem comes off easily “en bloc”. If one tries to pull the cerebellum without a large enough tentorium opening, it may break off (four cases). Nevertheless, our fixation method in most cases has shown itself to be safe for handling the whole brain.

### Post-fixation

This step is necessary to complete the fixation of the brain, particularly in the territory of the vertebro-basilar system. The post-fixation time can be as long as several years, provided it is stored at 4^°^C for most of the morphological techniques mentioned above. Although the time of post-fixation should be adapted to the specific needs of the study, in our method, we postfix the brain for 6 weeks and then photograph the brain and any of the blocks into which it can be divided. One important issue is overfixation of the brain. Although the concept of overfixation is rather vague and subjected to the use and preferences by different authors, in our protocol, we deem the period 6 weeks of postfixation in 4% paraformaldehyde reasonable to allow complete diffusion of the fixative in the brain. No detrimental effect in any of the techniques applied in the study was observed.

### *Ex vivo* MRI

We do not have direct experience of obtaining MRI images from the whole brain from donors immediately after the intravascular fixation of the brain. We can assume that, if adequate facilities were available, MRI *ex vivo*, *in situ*, and after fixation would be equally feasible.

The intracarotid perfusion of the brain has been reliably used to obtain MRI images that can be co-registered onto histology sections. This approach (nowadays widely used by us and others) provides the best anatomical correspondence to MRI images, to conduct several different determinations of brain parameters (Iglesias et al., 2018; Wisse et al., 2021a; Yushkevich et al., 2021; Alkemade et al., 2022). Previous work by our laboratory and others approximated histology and MRI images, not belonging to the same subjects, resulting in only a proxy for anatomical annotations of MRI images (Insausti et al., 1998; Franko et al., 2014; Delgado-Gonzalez et al., 2015, 2017). Our fixation protocol *shows* a far superior congruence of both histology and MRI compared to our previous work (Iglesias et al., 2018). Although its use has to date been restricted to the human temporal lobe, it could equally be extended to the whole hemisphere (DeKraker et al., 2021).

### Tissue deformation and shrinkage

One aspect that is particularly interesting is the issue of deformation of fixed human brains, especially when fixation is achieved by immersion. The jelly-like structure of the unfixed human or animal brain can result in deformation of the brain in uncontrolled ways. In some laboratories, the brain is suspended by the basilar artery during immersion fixation (usually 3–4 weeks), in which case the deformation is hypothetically reduced due to the rigidity of the brain conferred by penetration of the vessels. A less effective approach is resting the brains on a bed of soft cotton. Due to the intracranial relationships of the brain with the surrounding cerebrospinal fluid, intrinsic architecture of brain nuclei, white matter fascicles and vascular arterial and venous trees are largely intact, but many small changes are observable at the histological level. Therefore, the overall 3D architecture from reconstructions derived from intensive *ex vivo* imaging and histology may be suboptimal. Other more complex protocols have been described, all of which aim to preserve the appearance of the brain and the integrity of the structures in the 3D space of the human brain (Shatil et al., 2016; Maranzano et al., 2020). Fixation leads to a degree of brain shrinkage that may confound volumetric data when compared with *in vivo* MRI. Brain shrinkage, thus, needs to be taken into consideration. This is an old technical issue (Mouritzen Dam, 1979) that must be adjusted according to the different fixation methods used in different laboratories. All fixation protocols lead to some shrinkage, with perhaps the highest being up to 40% in paraffin embedded protocols (Quester and Schroder, 1997). Although we did not perform any specific analysis on the shrinkage of the brain, a recent study in immersion-fixed brain reported a shrinkage of 5%–7% in the whole brain volume, with no effect on the hippocampal histology (Annese et al., 2014). Anatomical analysis of the medial temporal lobe in tissue fixed in the same laboratory, and with the same technique suggest that the shrinkage in Annese et al. (2014) is very likely similar to what can be obtained by our method (Insausti et al., 2013).

### Immunoreactivity to different antibodies

Waldvogel et al. (2006) report a list of more than 15 different antibodies that display appropriate and specific labeling in human brain sections. It is important to note that the specificity and intensity of the labeling often depend on a previous titration study to determine the optimal concentration of the antibody for each specific immunoreaction, to get the lowest possible background and the best possible signal-to-noise ratio. We tried several antibodies in our material and concluded that the quality of the stain is comparable to—if not better than—images published in different reports. In this respect, our procedure offers no substantial advantage in individual labeling. However, our method provides a consistent quality of fixation and, in our experience, uniform immunohistochemistry using, for example, anti-Tau AT8 stain along 8–10 cm of tissue blocks, corresponding to the whole rostro-caudal extent of a complete human temporal lobe, in an extensive series of cases [Yushkevich et al. (2021), 21 cases]. This highlights the consistency of the method in terms of collecting larger series than in other studies [e.g., Bian et al. (2016), three cases; Beaujoin et al. (2018), one case; Alkemade et al. (2022), two cases]. The same holds true for its applicability to intracellular filling and electron microscopy. The quality of the intracellular filling and the level of detail, as far as the most delicate branches are concerned, are comparable to any other intracellular filling in experimental studies in rodents (Benavides-Piccione et al., 2020; Cano-Astorga et al., 2021).

As mentioned above, electron microscopy requires an excellent fixation, with the shortest postmortem interval possible. Most of the studies using electron microscopy in human brain tissue adapt to the condition of the brain tissue obtained. The fixation is critical, and in many cases, the brain tissue comes from biopsy studies (Lim et al., 1997; Cano-Astorga et al., 2021) or temporal lobectomies for the treatment of seizures (e.g., DeFelipe et al., 1993; Del Rio and DeFelipe, 1994; Arellano et al., 2004). In such cases, a rapid immersion in fixative allows a virtually non-existent PMI, and the small volume of tissue for fixation leads to excellent results. The obvious drawback is the very restricted brain region available to study and it should also be noted that crucial factors such as medical treatment and the pathological substrates of the patients may modify the brain tissue obtained after surgery.

## Conclusions

Fixation of the human brain is a challenging race against the clock to ensure that the structural preservation of brain tissue is as close as possible to living tissue. Regarding this limitation, it is difficult to reduce the time between death and fixation (even in experimental animals). Differing human circumstances, from personal to social and economic, mean that obtaining homogeneous conditions for the study is highly improbable. Indeed, most of the time, researchers must simply “make do” with whatever brain tissue they may get.

Our method represents an advance since it unifies—in a single method—human brain fixation through bilateral intracarotid access to flush the blood of the brain with saline and fixation with 4% paraformaldehyde in phosphate buffer. We provide evidence that our method is reliable, homogeneous, and uniform, with high validity for *ex vivo* MRI images of the brain, lacking distortion since the tissue is fixed intracranially. We deem this last point regarding distortion to be very important, particularly when this method needs to be combined with histological or immunohistochemical methods. Further studies are necessary to hone our method by introducing controlled variables such as the pressure of incoming fluids according to age, and reported vascular status, which—through the control of volume delivery—can be accounted for to improve the preservation of the original *in vivo* structural features of the human brain in control and pathological conditions.

## Data Availability Statement

The original contributions presented in the study are included in the article, further inquiries can be directed to the corresponding author/s.

## Ethics Statement

The studies involving human participants were reviewed and approved by University Ethical Committee, Albacete, Spain. The patients/participants provided their written informed consent to participate in this study.

## Author Contributions

RI and EA-P contributed to study concept and design. All authors contributed to the acquisition analysis and interpretation of data. LW, SR, JD, EA-P, PY, and RI contributed to the draft of the manuscript. All authors drafted/revised the manuscript for content. All authors contributed to the article and approved the submitted version.

## Footnotes

^1^The *ex vivo*, *ex situ* perfusion employed previously consisted of the exposure of the circle of Willis and identification of the carotid arteries. One of them was ligated (the side depending on the presence of arteriosclerotic plaques in the arterial wall), while a cannula was inserted in the other side. The basilar artery was also ligated at the level of the pons. Alternatively, one carotid artery and the basilar artery were cannulated. In this case, the fixative flow is directed to the other hemisphere through the anterior communicating artery. It must be checked the presence of both communicating arteries, anterior and posterior, without which the intravascular perfusion of the brain would be incomplete (the Willis polygon presents one of the highest rates of variability). Once the ligatures were in place, the brain was flushed with saline and then with the fixative.

^2^The hippocampal commissure contains hardly any hippocampal projections crossing to the other side other than some presubicular and entorhinal cortex projections (Amaral et al., 1984; Demeter et al., 1985).

^3^There are also other methods of body procurement (purchasing, etc.) but in those cases the PMI may preclude the use of the present method.
